# Effects of tracer position on screw placement technique in robot-assisted posterior spine surgery: a case–control study

**DOI:** 10.1186/s12891-023-06547-y

**Published:** 2023-05-25

**Authors:** Meng Yi, Jipeng Song, Yao Zhang, Wancheng Lin, Mingtao Yao, Yuyu Fan, Lixiang Ding

**Affiliations:** Department of Spine, Beijing Shijitan Hospital, Capital Medical University, No.10 Tieyi Road, Yangfangdian, Haidian District, Beijing, 100038 People’s Republic of China

**Keywords:** Robot-assisted posterior spine surgery, Screw Accuracy, Tracer Location

## Abstract

**Introduction:**

Robot-assisted spine surgery is increasingly used in clinical work, and the installation of tracers as a key step in robotic surgery has rarely been studied.

**Objective:**

To explore the potential effects of tracers on surgical outcomes in robot-assisted posterior spine surgery.

**Methods:**

We reviewed all patients who underwent robotic-assisted posterior spine surgery at Beijing Shijitan Hospital over a 2-year period from September 2020 to September 2022. Patients were divided into two groups based on the location of the tracer (iliac spine or vertebral spinous process) during robotic surgery and a case–control study was conducted to determine the potential impact of tracer location on the surgical procedure. Data analysis was performed using SPSS.25 statistical software (SPSS Inc., Chicago, Illinois).

**Results:**

A total of 525 pedicle screws placed in 92 robot-assisted surgeries were analyzed. The rate of perfect screw positioning was 94.9% in all patients who underwent robot-assisted spine surgery (498/525). After grouping studies based on the location of tracers, we found there was no significant difference in age, sex, height and body weight between the two groups. The screw accuracy (*p* < 0.01)was significantly higher in the spinous process group compared to the iliac group (97.5% versus 92.6%), but the operation time (*p* = 0.09) was longer in comparison.

**Conclusion:**

Placing the tracer on the spinous process as opposed to the iliac spine may result in longer procedure duration or increased bleeding, but enhanced satisfaction of screw placement.

## Introduction

In recent years, intraoperative navigation techniques have been widely applied in spine surgery. With the development of robotics in spine surgery, robot-assisted pedicle screw placement has exhibited major advantages over traditional non-robotic techniques, including higher accuracy and safety [1–5].

The robotics adopts an anchored tracer device to establish a fixed connection between the optical tracking system and the surgical area, to present 3-D images in front of the surgeon at a scale size, which allows individualized surgical screw planning and simulated staple placement for different patients, as well as enables screw placement in a shorter time with the assistance of a robotic arm. However, in practice, the tracer needs to be installed in an area that remains relatively fixed, most commonly either in the spinous process or posterior superior iliac crest, or to the surgical bed. However, the following problems [6] should be noted in the placement of tracers: (1) difficulty in placement of tracers in obese patients, inability to adequately expose the surgical area after placement of tracers; (2) Tracer placement intersects with surgical instruments, affecting surgical operations; (3) changes in the tracer and the original planned anatomy after correction of the deformity, leading to deviations in subsequent screw placement [7–9]. At present, there is no definitive conclusion as to which method is superior for tracer placement.

The purpose of this study is to investigate the effects of tracer positions on robot-assisted posterior spinal surgery and to explore an optimal tracer position to support the achievement of robot-assisted clinical practice.

## Method

We reviewed all patients who underwent robotic-assisted spine surgery at Beijing Saitan Hospital over a 2-year period from September 2020 to September 2022. And for the medical records we had, we screened patient-related information according to inclusion and exclusion criteria.

Inclusion criteria were thoracolumbar spinal stenosis, disc herniation, fracture, and lumbar spondylolisthesis. Exclusion criteria were severe spinal infection, primary and secondary malignancy of the spine, previous surgery in the same location (revision surgery), and incomplete demographic or clinical information. Tracer placement was randomly selected preoperatively. All patients were divided into sphenoid group and iliac crest group according to the specific placement location. This study was approved by the Ethics Committee of Beijing Shijitan Hospital, Capital Medical University. sjtkyll-lx-2023(022) The preoperative informed consent was obtained from each participant.

All methods were carried out in accordance with relevant guidelines and regulations or declaration of Helsinki.

### Robot-assisted surgery system

**Fig. 1:**
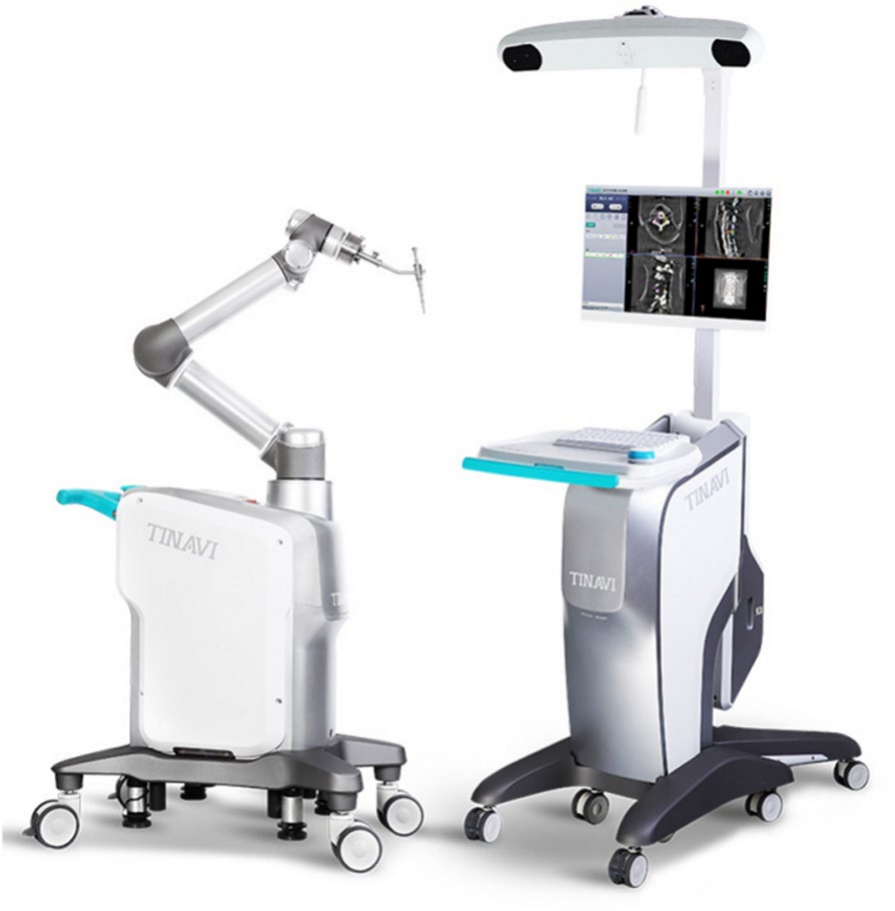
The TianJi Robot system is composed of a robotic workstation, an optical tracking system, and a robotic arm

The robotics is a navigation and positioning device integrated a robotic arm, tracker, and workstation, which has been developed by Beijing TINAVI Medical Technologies (Fig. 1). The C-arm machine used in conjunction with the robot can perform an overall scan of the patient. The surgical robot accurately locates the patient's structures based on the position of the tracer and delivers the 3D model to the operating table. The surgeon can perform a simulated screw placement in the operating table. After planning all screw positions, the robot's operating arm automatically adjusts the position and the surgeon can accurately drive the screws into the desired position.

### Outcome assessment

Relevant information was collected as follows: (1) age, sex, and BMI; (2) surgical medical team (2 senior and 1 attending physicians), diagnosis, operative time, operative bleeding, number of screw placements, mean screw placement time, and mean segmental bleeding; (3) overall screw placement accuracy, screw placement satisfaction (A + B)/(A + B + C + D) × 100%, postoperative hospitalization time(Time between the day of surgery and when the patient can resume free movement), and complication rate.

### Surgery method

**Fig. 2:**
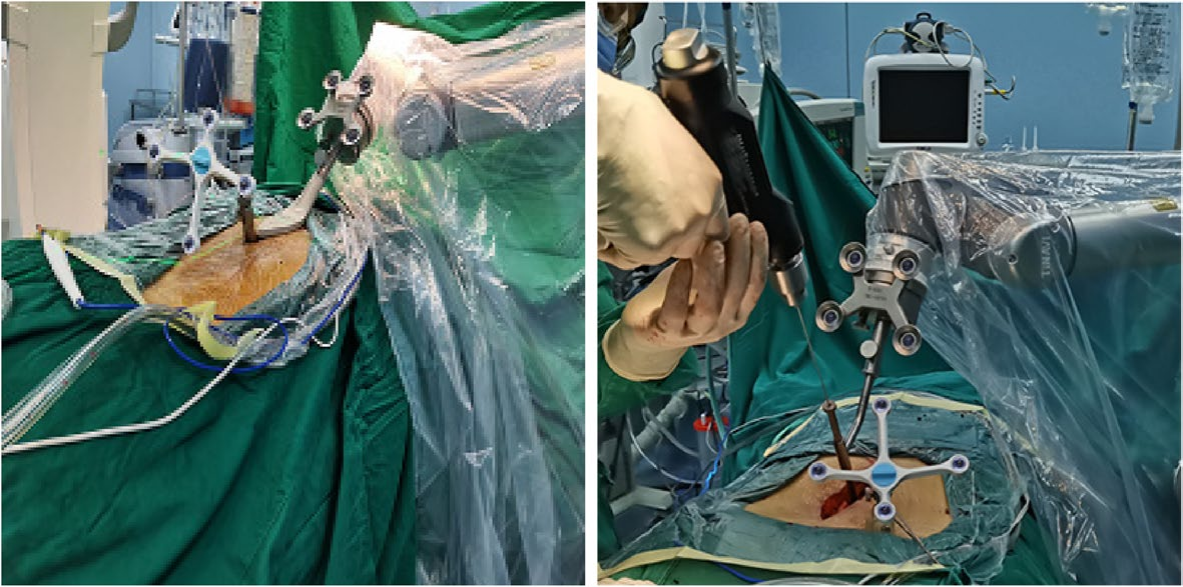
Left: The tracer is mounted on the spinous process of the adjacent surgical segment; Right: The tracer is mounted on the posterior superior iliac sipne

**Fig. 3:**
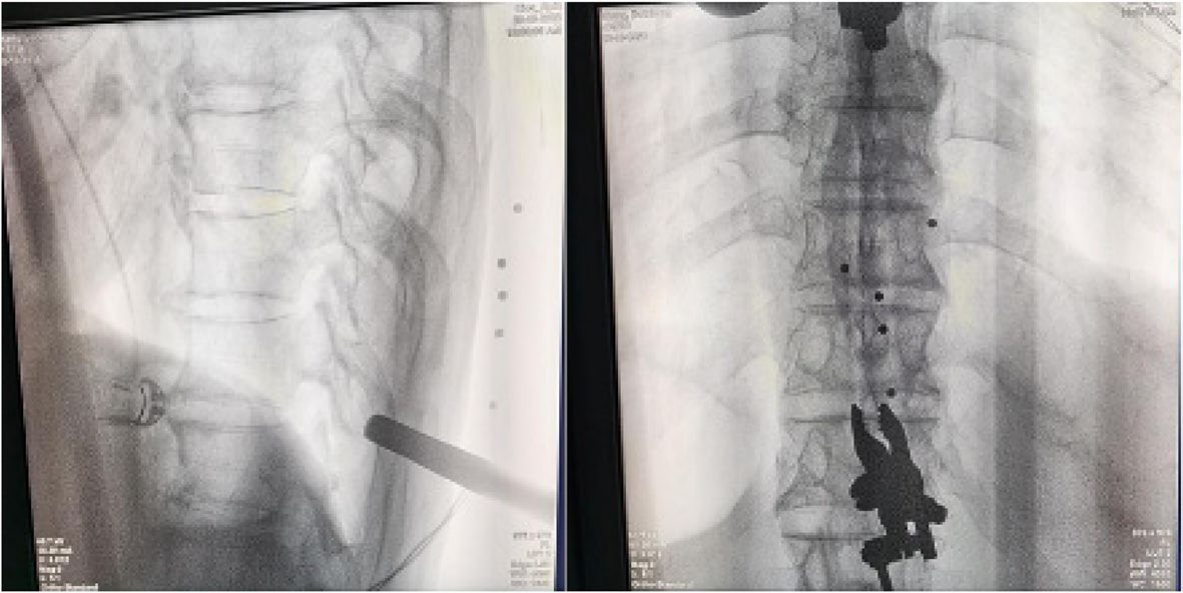
Fluoroscopy shows that the 5 points in the scale are completely within the operative area

A patient was placed prone on the spinal bed after general anesthesia. A suitable posterior median incision was made. The skin, subcutaneous, and supraspinous ligaments were incised layer by layer. The sacrospinous muscle was dissected along both sides of the spinous process according to the pre-existing CBT or PS pathway. After the exposure was completed, the gauze was filled and compressed to stop bleeding. The tracer was mounted according to a pre-planned installation. The tracer could be placed in 3 ways. The two most common ways were: (1) Mounting and fixing the tracer to the posterior superior iliac crest using 2 kerf pins; (2) Fixing the tracer to the spinous process of a segment adjacent to the operated segment using a spinous clamp (Fig. 2). After installation, the robot arm was connected to the guide (scale) and moved to the surgical area. A perfect installation was considered when the fluoroscopic front and side positions indicated that 5 points in the scale were completely within the range (Fig. 3). The C-arm fluoroscope was used to scan the entire surgical section in three dimensions. The images were transferred to the operating table, where the technician and the surgeon worked together to simulate the placement of the screw. We performed a re-fluoroscopy to initially confirm that the screws were in good position and then sutured the wound.

### Accuracy analysis

**Fig. 4:**
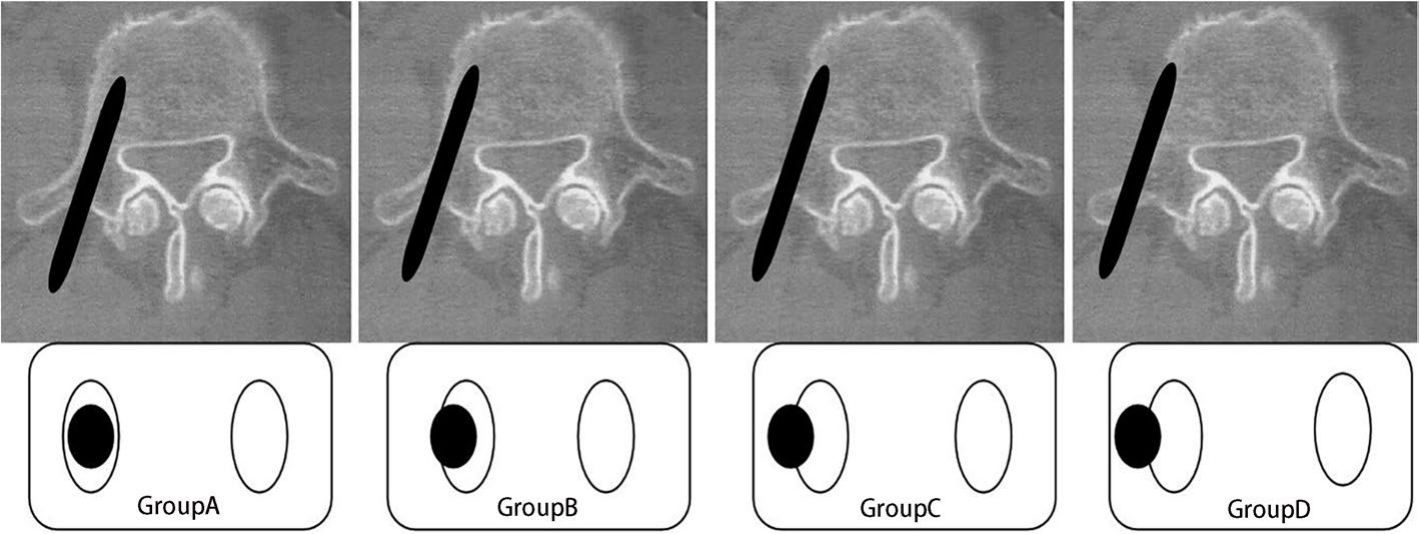
Modified Gertzbein-Robbins scoring system

Imaging data were evaluated jointly by a senior surgeon and a radiologist. The intraoperative planning imaging data were evaluated along with the postoperative review of CT imaging data to assess the reasonableness and accuracy of screw path. A modified Gertzbein-Robbins classification was adopted to record the accuracy of each screw. The degree of deviation was classified according to the distance from the edge of the pedicle as: Grade A (completely within the pedicle), Grade B (breach ≤ 2 mm), Grade C (2 mm < breach ≤ 4 mm), and Grade D (breach > 4 mm) (Fig. 4). The direction of deflection was recorded. Grades B-D indicated "Malposition". Minor deviations of less than 2 mm were clinically acceptable, while Grades C to D were defined as significantly malpositioned [10].

### Statistical methods

Data analysis was performed using SPSS.25 statistical software (SPSS Inc., Chicago, Illinois). Continuous variables were described by means and standard deviations (SD), while categorical variables were described by frequencies and percentages. Differences between groups were analyzed using chi-square tests. Student's t-test was used for the comparison of two normally distributed data. Categorical variables were assessed using chi-square tests or Fisher's exact tests. A *p* < 0.05 was considered a statistically significant difference.

## Results

A total of 92 patients were included in this study, including 49 males and 43 females. These patients were diagnosed with thoracic spinal stenosis (*n* = 40), thoracolumbar fracture (*n* = 30), slipped spine (*n* = 20), or scoliosis (*n* = 2). The mean height was 1.65 ± 0.07 m (range: 1.5–1.8 m). The mean body weight was 71.17 ± 13.20 Kg (range: 35–107 Kg). The mean BMI was 26 ± 3.82 Kg/m^2^ (range: 13.5–33.79 Kg/m^2^). All procedures were performed by 3 different groups of spine surgeons. Each surgical team included 2 senior- and 1 intermediate-rank surgeons, who were skilled in robotic surgery.

A total of 525 screws were inserted with robotic assistance, of which 498 achieved clinically satisfactory results (Grades A + B), with a satisfaction rate of 94.9%. All patients were divided into spinous process group and lliac spines group. The lowest satisfaction rate was found in the patients with scoliosis in spinous process group. A total of 12 screws were placed, 10 screws achieving grade A/B, and two screws in unsatisfactory positions. The patients had no associated complications.

The average time spent in robot-related operations was 43.26 ± 22.91 min. The duration included the entire process of establishing connection, scanning, simulating the placement of the screw, transferring holes, and driving the screw. The mean surgical bleeding (mean segment) was 112.16 ± 88.86 ml. The most bleeding of 384 ml occurred in thoracolumbar segment fractures with multi-segmental spinal stenosis. The least mean bleeding occurred in a patient with scoliosis who underwent only spinal screw placement orthosis without laminar spinal decompression. The average postoperative time to discharge was 12 days. The longest hospital stay was extended to 31 days due to infection. Postoperative complications such as surgical site infection, broken screws and rods, and unexplained pain in the surgical area were observed in 5 cases. These included three incisional infections that improved with subsequent debridement, one surgical site pain that improved with epidural glucocorticoid injections and one revision surgery due to displacement of the internal fixation device. The overall incidence of complication was 4.6%.

Of the 92 patients who participated in the trial, 45 patients were in the spinous process group and 47 patients were in the iliac spine group. Among the patients in the spine group, there were 25 males and 20 females. Their mean age was 66.62 ± 2.36 years, mean height was 1.62 ± 0.01 m, mean weight was 72.47 ± 1.62 Kg, and the mean BMI was 26.21 ± 0.38 Kg/m^2^. 24 male patients and 23 female patients, 47 in total, belonged to the iliac spine group. The basic information of this group of patients showed a mean age of 65.02 ± 2.35 years, a mean height of 1.64 ± 0.01 m, a mean weight of 69.93 ± 22.21 Kg, and a mean BMI of 25.81 Kg/m^2^. Among the patients in the spinous processs group, 22 were diagnosed with lumbar spinal stenosis, 12 with fractures, 10 with spondylolisthesis, and 1 with scoliosis. In the other group, there were 18 patients with spinal stenosis, 18 patients with fractures, 10 with spondylolisthesis, and 1 with scoliosis.

There were no significant differences between the two groups in age, sex, height, body weight, BMI, clinical diagnosis, and surgery-related indicators. Intraoperative operating time (including tracer placement, surgical planning, navigation-controlled screw placement) was comparable (*p* = 0.09). There might be differences in patients with different diagnoses requiring additional decompression or with different levels of surgical ease. No significant differences were observed in the mean bleeding(128.80 ± 15.20 ml vs 96.23 ± 10.47 ml), postoperative hospital stay(12.49 ± 1.12 days vs 12.81 ± 0.80 days), or complication rates between the two groups. In terms of accuracy of screw placement, the spinous process group appeared to have more satisfactory screw placement (97.5% vs 92.6%, *p* ＜ 0.01) (Table 1). There were no significant differences among the three surgical teams (*p* = 0.68). There might be relatively more surgical complications in teams 1 and 2. However, there were relatively more surgical complications in team 2. There was no statistically significant difference in the incidence of complications.

**Table 1:** Differences of baseline and surgical characteristics between two groups

Index	Spinous process group (*N* = 45)	Iliac spine group (*N* = 47)	*p*
Age (year)	66.62 ± 2.36	65.02 ± 2.35	0.63
Sex (male/female)	25/20	24/23	0.67
Height (m)	1.65 ± 0.01	1.64 ± 0.01	0.28
Weight (Kg)	72.47 ± 1.62	69.93 ± 2.21	0.36
BMI (Kg/m^2^)	26.21 ± 0.38	25.81 ± 0.69	0.62
Diagnosis			
Spinal stenosis	22	18	
Fracture	12	18	
Spondylolisthesis	10	10	
Scoliosis	1	1	0.24
Operating time (min)	47.45 ± 3.99	39.26 ± 2.60	0.09
(Planning and screw setting)			
Bleeding volume (ml)	128.80 ± 15.20	96.23 ± 10.47	0.08
(Single segment)			
Screw satisfaction	97.50%	92.60%	< 0.01*
(A + B)/(A + B + C + D) × 100%			
Length of hospital stay after surgery (day)	12.49 ± 1.12	12.81 ± 0.80	0.82
Complications			
Infection	2	4	
Revision	0	1	0.41
Medical group			
Group.1	20	17	
Group.2	15	19	
Group.3	10	11	0.68

*p < 0.01

## Discussion

The advent of robot-assisted technology has simplified spinal surgery [5, 11]. The surgeon can plan screw path on the robotic table, select the screw size in the model, and then simply the transfer of kerf pins and screws into the intended position after the robotic arm has completed movement according to the plan. The difficulty of learning curve lies in installation of a reliable tracer and development of a reasonable screw trajectory, as well as complete scanning of surgical site image in the operating table. This is necessary to ensure that the tracer does not affect the operation as much as possible. Inappropriate tracer can lead to displacement of all screws and even irreversible nerve and vascular damage [5, 8].

The literature had reported dissatisfaction rates of up to 40% for screws with the freehand arch screwing technique, while satisfaction rates of 91% to 100% could be achieved with robot-assisted screw placement [1]. In our study, overall screw placement satisfaction rate (modified Gertzbein-Robbins scale Grades A + B) was 94.9%. Even in the relatively low-satisfaction iliac spine group, a screw satisfaction rate of 92.6% was achieved. Robotic-assisted accuracy was higher than that of the freehand screw placement [2, 12, 13].

In terms of postoperative complications, especially the revision rate, was lower in robotic-assisted surgery than conventional surgery (0.58–1.7% vs. 2–5%) [14–17]. In our study, the incidence of postoperative complications was 0.076%. Only one patient underwent revision surgery due to severe osteoporosis that led to displacement of the internal fixation device and compression of the nerve after 3 months. Intraoperative bleeding appeared to be greater in the spinous process group than in the iliac spine group, which may be related to displacement of the tracer in the spinous process group. It is customary to place the tracer on the spinous process adjacent to the operative segment and to expose the paravertebral muscles of the segment where the tracer is located. Thus, sufficient space can be obtained for the tracer, to avoid interference with the surrounding tissue, or bleeding due to stripping of soft tissue. The greater the tension of the paravertebral muscles, the more likely the screw will be deflected during screw placement [3, 18]. For the iliac spine group, the tracer is fixed in the posterior superior iliac crest by two kerf pins, which is relatively less invasive. We worried about the pain associated with installing the tracer in the iliac spine, similar to the complications associated with taking autogenous bone from the iliac bone. In practice, few patients complain of discomfort in this location. Thus, tracers in the iliac spine position are more stable and easier to install than those on the spinous process [19]. However, no difference was observed in stability. We tried to avoid touching the tracer during the procedure [12].

Compared to tracer placement on the iliac spine, screws in the spinous process group have shown higher postoperative screw placement satisfaction. However, there is no definitive conclusion regarding the advantages and disadvantages of different tracer placements. There have been articles reporting on a 3d navigation device that adheres to the skin in the surgical area, but the effect does not compare convincingly with that of an external tracker [7]. The more popular international practice is to place the tracer in the spinous process. The accuracy of screw placement is related to the distance of the tracer. A distance greater than three segments may lead to deviation of the screw. For surgical regions such as thoracic and the upper lumbar spine, tracer placement at the iliac spine resembles the hypotenuse of a triangle. However, the spinous process of adjacent segments is closer to the surgical region, as we always choose the adjacent surgical segment or the next adjacent segment as the target location for tracer placement. We noticed that tracer placement on the spinous process may correlate with the operation time. Although there was no statistically significant difference between the two groups, in practice, tracer placement on the spinous process increased the chance of intersection between the arm and the sleeve, especially for the adjacent tracer segments. This intersection could affect the movement of the arm to the intended position. If the tracer is displaced, the entire 3D image stored in the operating table is more or less biased, which can cause misalignment of subsequent screws. This is quite dangerous for the cervicothoracic segment with a narrower pedicle. In addition, intraoperative rescanning and replanning can occur during the procedure due to the mechanical arm and tracer interfering with each other. Interference results in a significant increase in operating time and in radiation exposure to the patient. In such cases, many surgeons abandon the use of robots, in favor of freehand screw placement. The probability of cortical invasion by the pedicle screw is greater than 20.6% when the tracer is more than 3 segments away from the operated segment whereas only 4.8% when there is only 1 segment [3, 18]. When the tracer is placed far away or through an unstable area during the procedure, slight movement signal may not be fully transmitted to the missing device, resulting in failure to detect this abnormal activity and thus affecting the accuracy of screw placement. It is therefore necessary to start screw placement process from a position far from the tracer, to reduce the probability of tracer displacement. In patients with severe scoliosis, especially during correction of complex thoracic bending deformities, the accuracy of screw placement can seriously affect neurological functions. Thus, the tracer should be placed as close to the operating level as possible, and even a segmental scanning screw placement is acceptable. Although this process increases the operating time, the accuracy of screw placement can be better grasped, requiring the surgeon to weigh the pros and cons [12, 20].

Some limitations of this study should be mentioned. Firstly, this is a single-center, case–control study. In the future, multicenter, large sample-size study will be performed to validate the advantages and disadvantages of the two different tracer mounting methods. Secondly, we excluded those cases in which the screws were placed manually due to intraoperative displacement of missing device or poor path design. Thirdly, there were varying degrees of selection bias regarding tracer installation. Finally, we did not fully determine which method of tracer installation was chosen for row procedure.

## Conclusion

The robot-assisted pedicle screwing technique has significantly improved screw accuracy compared to the traditional freehand screwing technique. Placing the tracer on the spinous process as opposed to the iliac spine may result in longer procedure times or increased bleeding, but significantly increased satisfaction of screw placement.

## Data Availability

All data used by or generated in this study is available from the corresponding author upon reasonable request.
